# Variation Analysis of Physiological Traits in *Betula platyphylla* Overexpressing *TaLEA-ThbZIP* Gene under Salt Stress

**DOI:** 10.1371/journal.pone.0164820

**Published:** 2016-11-01

**Authors:** Xiyang Zhao, Tangchun Zheng, Longting Shao, Zhenhai Xiao, Fuwei Wang, Shuchun Li, Lina Zang, Mi Zheng, Ying Li, Guan-Zheng Qu

**Affiliations:** 1 State Key Laboratory of Tree Genetics and Breeding (Northeast Forestry University), School of Forestry, Northeast Forestry University, Harbin 150040, China; 2 Beijing Key Laboratory of Ornamental Plants Germplasm Innovation & Molecular Breeding, National Engineering Research Center for Floriculture, Beijing Laboratory of Urban and Rural Ecological Environment, Key Laboratory of Genetics and Breeding in Forest Trees and Ornamental Plants of Ministry of Education, School of Landscape Architecture, Beijing Forestry University, Beijing, 100083, China; 3 Tree Seedling Management Station, Forestry Department of Jilin Province, Changchun 130000, China; Pennsylvania State University, UNITED STATES

## Abstract

The aim of this study was to determine whether transgenic birch (*Betula platyphylla*) ectopic overexpressing a late embryogenesis abundant (*LEA*) gene and a basic leucine zipper (*bZIP*) gene from the salt-tolerant genus *Tamarix* (salt cedar) show increased tolerance to salt (NaCl) stress. Co-transfer of *TaLEA* and *ThbZIP* in birch under the control of two independent CaMV 35S promoters significantly enhanced salt stress. PCR and northern blot analyses indicated that the two genes were ectopically overexpressed in several dual-gene transgenic birch lines. We compared the effects of salt stress among three transgenic birch lines (L-4, L-5, and L-8) and wild type (WT). In all lines, the net photosynthesis values were higher before salt stress treatment than afterwards. After the salt stress treatment, the transgenic lines L-4 and L-8 showed higher values for photosynthetic traits, chlorophyll fluorescence, peroxidase and superoxide dismutase activities, and lower malondialdehyde and Na^+^ contents, compared with those in WT and L-5. These different responses to salt stress suggested that the transcriptional level of the *TaLEA* and *ThbZIP* genes differed among the transgenic lines, resulting in a variety of genetic and phenotypic effects. The results of this research can provide a theoretical basis for the genetic engineering of salt-tolerant trees.

## Introduction

Birch (*Betula Platyphylla*) is one of the most extensively distributed broadleaf species in the northern and southwestern forested areas of China [1]. Because of its excellent wood quality, birch is widely used in the production of paper, furniture, and plywood [2]. Previous studies on birch have focused on its breeding [3–5], fiber length [6], fungi in bark [7, 8], genetic transformation [9], and molecular markers [10, 11].

Soil salinity, which is a major abiotic stress that reduces plant productivity, affects large areas around the world [12]. In China, the total area of saline-alkali soil is approximately 8.11 × 10^7^ ha, or approximately 8%-9% of the total land area [13]. Salinity has been shown to have substantial effects on plant growth and development. The osmotic stress and ion toxicity associated with saline soils result in low plant yields and negatively affect the growth of agricultural and forest crops [14]. To ensure both their own survival and that of their offspring, plants have developed a range of strategies, including gene expression regulation, to cope with adverse conditions through various physiological adaptations [15].

Late embryogenesis abundant (LEA) proteins were first discovered in germinating cotton (*Gossypium hirsutum*) seeds [16], and *LEA* genes were subsequently found to be one of the most important stress-associated gene families. Many studies have demonstrated that *LEA* genes are associated with tolerance against salt and other stresses [17–19]. Basic leucine zipper (bZIP) proteins comprise one of the largest transcription factor families in plants [20]. bZIP transcription factors are involved in plant defense, plant senescence, responses to various environmental stresses, and developmental processes [21]. One of the bZIP protein families related to stress responses is the TGA family, whose members regulate the expression of some stress-responsive genes [22].

Although birch has a strong cold resistance, it is weak in salt tolerance, which limits the popularization and application of birch in saline soils. In order to gain transgenic birch with salt tolerance, both *TaLEA* and *ThbZIP* genes from *Tamarix* were transformed into birch and then subjected to salt stress treatments. The aim of these experiments was to determine whether these genes affected salt tolerance, and to detect the variation in physiological characters among the different transgenic lines. This information will provide a theoretical basis for molecular breeding in birch.

## Materials and Methods

### Construction of plant expression vector and plant transformation

**Fig 1:**
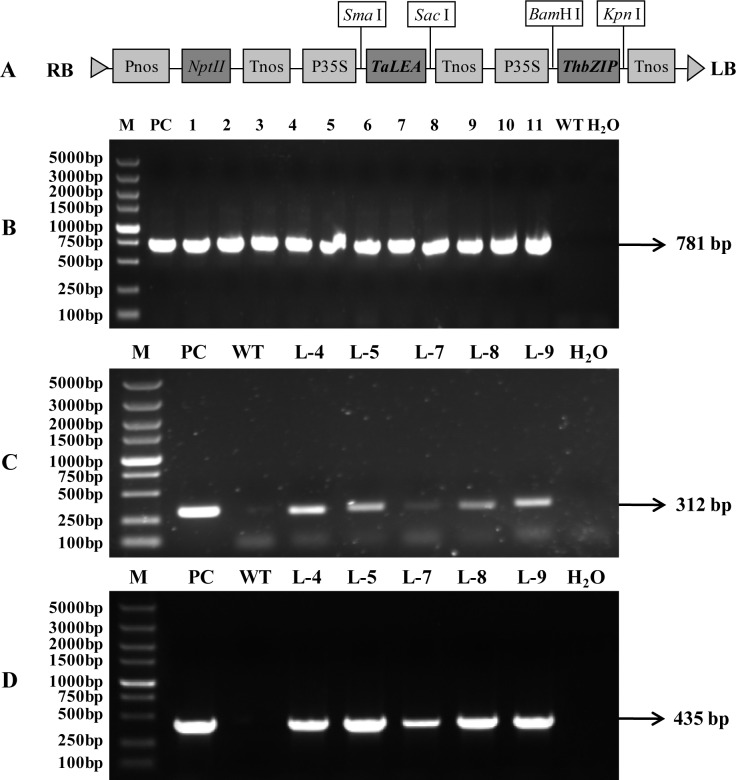
Map of the T-DNA construct and identification of overexpressing *TaLEA* and *ThbZIP* transgenic birches with PCR. (A) Schematic of the T-DNA region of the binary vector pROKII-*TaLEA*-*ThbZIP*. RB, right border; Pnos, nopaline synthase promoter; *NptII*, kanamycin resistance gene; Tnos, nopaline synthase terminator; P35S, CaMV 35S promoter; *Sma* I, *Sac* I, *Bam*H I, and *Kpn* I, four different restriction enzyme sites; *TaLEA*, *TaLEA* gene; *ThbZIP*, *ThbZIP* gene; LB, left border. Agarose gel electrophoresis of PCR products from wild type and transgenic lines with the primer of *NptII* (B), *TaLEA* (C), *ThbZIP* (D). M, DNA marker; PC, positive control; 1–11, eleven Km resistant lines; L-4, 5, 7, 8, 9, five transgenic lines both containing *TaLEA* and *ThbZIP* genes; WT, wild type plantlet; H_2_O, double-distilled water as negative control.

The *TaLEA* and *ThbZIP* genes were firstly cloned from *Tamarix* as described by Wang [23, 24]. Each individual gene was constructed into a pROK2 vector, respectively. Then the target fragment (P35S-*TaLEA*-Tnos) amplified from a pROK2-*TaLEA* vector was inserted into the pROK2-*ThbZIP* vector, concrete steps of which were described in detail in our previous research (Fig 1A) [25].

To study the functions of the *TaLEA* and *ThbZIP* in birch, transgenic birch *(B*. *platyphylla* Suk.) plantlets were obtained by *Agrobacterium*-mediated transformation. To prepare the infection liquid, an *Agrobacterium* culture was incubated until the OD_600_ was 0.6–0.8, then centrifuged at 3000 r min^−1^ for 5 min, finally, the collected pellets were diluted with sterile water to a final concentration of OD600 = 0.1. Leaves from 60-d-old clones were cut in half and the pieces were gently shaken in the infection liquid for 5 min. Then, the leaves were removed and excess liquid was absorbed with sterile filter paper. The infected birch leaves were then co-cultured on antibiotic-free differentiation medium (WPM medium supplemented with 20 g L^−1^ sucrose, 0.02 mg L^−1^ 1-naphthylacetic acid [NAA], 0.8 mg L^−1^ N_6_-benzyladenine [6-BA], 0.5 mg L^−1^ gibberellin [GA_3_], and 8 g L^−1^ agar) at 25 ± 2°C in the dark for 2 d. To eliminate bacteria, the co-cultured leaves were washed with 200 mg L^−1^ cephalosporin solution for 3–5 min. The excess liquid was absorbed with sterile filter paper, and then leaves were cultured in new differentiation medium (WPM medium supplemented with 20 g L^−1^ sucrose, 0.02 mg L^−1^ NAA, 0.8 mg L^−1^ 6-BA, 0.5 mg L^−1^ GA_3_, 8 g L^−1^ agar, 40 mg L^−1^ Km, and 500 mg L^−1^ cephalosporin). In the first week, bacteria elimination was performed every 2 d; subsequently, bacteria were eliminated every 7 d until small buds differentiated. The resistant buds grew into leaves, which were cut off and cultured on a differentiation medium containing antibiotics. Finally, adventitious shoots were transferred onto medium (WPM medium supplemented with 20 g L^−1^ sucrose, 1.0 mg L^−1^ 6-BA, and 8 g L^−1^ agar) to allow shoot growth for 2 weeks. To induce rooting from the shoots, 2-cm shoot cuttings were transferred to rooting medium (1/2 MS medium containing 20 g L^−1^ sucrose, 0.02 mg L^−1^ NAA, and 8 g L^−1^ agar).

### Detection of transgenic plantlets

Total DNA was extracted from all transgenic and WT birch lines using a modified CTAB method [26]. Using the extracted DNA as a template, the transformants were confirmed by polymerase chain reaction (PCR) amplification with specific primers for *NptII* (Kanamycin resistance gene), *TaLEA* and *ThbZIP* genes (S1 Table). The vector pROK2-*TaLEA*-*ThbZIP* served as the positive control, and WT birch and water served as the two negative controls. The PCR reactions were conducted using *ExTaq* DNA polymerase (TaKaRa, Dalian, China) according to the manufacturer’s protocol. The cycling conditions were as follows: pre-denaturing for 3 min at 94°C followed by 35 cycles of denaturing at 94°C for 30 s, annealing at 58°C for 30 s, and extension at 72°C for 1 min, and a final extension at 72°C for 7 min. The PCR products were detected on 1.0% agarose gels.

To detect *TaLEA* and *ThbZIP* in birch, total RNA from WT and transgenic birch clones was isolated as described by Qu [27]. Subsequently, 10 μg of total RNA was separated on a 1% agarose-denaturing formaldehyde gel, transferred to a Hybond-N^+^ nylon membrane, and fixed with UV cross-linking (254 nm, 8 min) for the northern blot analysis. The membrane was hybridized with full-length *TaLEA* and *ThbZIP* genes labeled with DIG-dUTP. Hybridization and detection were using a DIG Northern starter Kit,(Roche, Basel, Switzerland).

### Salt tolerance analyses of transgenic birch in tissue plantlets

To test the salt stress tolerance of transgenic birchs, the shoot of tissue cultured seedlings was cut into 1- cm pieces and cultured on WPM differentiation medium containing 2 g L^−1^, 4 g L^−1^ or 6 g L^−1^ NaCl. Moreover, the wild-type and the transgenic plants exhibiting similar height (about 3 cm in length) were grown on 1/2 MS medium supplemented with 4 g L^−1^ or 6 g L^−1^ NaCl for rooting. The growth condition was controlled at 25°C in a 16 h light/8 h dark photoperiod at an intensity of ~2000 lux. The phenotypes of seedlings were photographed and measured after 20 d of growth.

### Salt stress experiment in greenhouse

For growth comparison of plants in soil, three transgenic birch lines (L-4, L-5, and L-8) and one wild type line (WT) were used in this study. In April 2014, the four lines were propagated and grown in separate pots in a greenhouse. After 60 d, 200 healthy plants in each line were selected as the experimental materials (about 40 cm in height). The greenhouse was controlled at a relative humidity of 65–75% with an average temperature of 27 ± 2°C. Cool white fluorescent lights supplied photons at 200 μmol m^−2^ s^−1^.

Thirty uniform WT seedlings were selected and divided into five group as the preliminary material, One group was designated as the control group and the remaining four groups were treated with NaCl at various concentrations (2, 4, 6, and 8 g L^−1^). NaCl solutions were applied at 18:00–19:00 every 2 d for 16 d. The phenotypes of each group were observed and instantaneous net photosynthesis rate (Pn) and chlorophyll fluorescence parameters (F_v_/F_m_) were measured at 0, 2, 4, 6, 8, 10, and 12 d during the stress treatment. Pn values were measured from 8:30 a.m. to 11:30 a.m. with a Lico-6400 portable photosynthesis measuring system (Li-cor Inc., Lincoln, NE, USA) on the third to fifth fully expanded leaves of each plant. The conditions during photosynthetic trait measurements were as follows: leaf temperature, 28°C; PPFD, 1400 μmol m^-2^ s^-1^; relative humidity, 60%; ambient CO_2_ concentration, 400 μmol mol^-1^. Chlorophyll fluorescence parameters were measured with the same leaves using a pulse amplitude modulation chlorophyll fluorometer MINI-PAM2500 (Walz, Effeltrich, Germany). Minimal fluorescence, F_0_, was measured in 30-min dark-adapted leaves using weak modulated light of < 0.15 μmol m^-2^ s^-1^. Maximal fluorescence, F_m_, was measured after an 0.8-s saturating white light pulse (6000 μmol m^-2^ s^-1^) in the same leaf with 2.9. Maximal variable fluorescence (F_v_ = F_m_–F_0_) and the photochemical efficiency of PSII (F_v_/F_m_) for dark-adapted leaves were calculated.

We selected 4 g L^−1^ NaCl as the most appropriate concentration based on the results of the preliminary experiment. One hundred uniform plants of each line (WT, L-4, L-5, and L-8) were selected as the experimental materials. All plants were watered with a 4 g L^−1^ NaCl solution every 2 d, and the Pn-photosynthetic photon flux density (PPFD) curves and Pn-CO_2_ concentration in air (Ca) curves were measured and analysesed by the method of Zhao [28] on 8 d of the salt treatments. Light saturation point (LSP), light compensation point (LCP), CO_2_ saturation point (CSP) and CO_2_ compensation point (CCP) were evaluated by fitting the data to the model function as follows:

$$Y=b_{0}+b_{1}X+b_{2}X^{2}$$ (1)

where *Y* is the Pn value, *X* is the PPFD (or Ca), *b*_0_ is a constant, and *b*_1_ and *b*_2_ are coefficients.

The CO_2_ saturation point (CSP) and CO_2_ compensation point (CCP) were evaluated by fitting the data to the model function.

Measurements of photosynthetic parameters, antioxidant enzyme activity, malondialdehyde (MDA) and Na^+^ contents were conducted on 0, 4, 8, 12, and 16 d of the salt stress treatment, using 10 plants from each line. Also the third to fifth fully expanded leaves of each plant were used for photosynthetic parameters, chlorophyll fluorescence parameters, antioxidant enzyme activity and Na^+^ contents assays. Photosynthetic parameters (Pn, intercellular CO_2_ concentration (Ci), stomatal conductance (Gs), and transpiration rate (Tr)) were measured by lico-6400, chlorophyll fluorescence parameters (F_v_/F_m_) were measured by MINI-PAM2500, the methods were the same with as ahead. The total superoxide dismutase (SOD) activity was assayed as described by Giannopolitis [29], total peroxidase (POD) activity was assayed as described by Rao [30], Malondialdehyde (MDA) was assayed as described by Heath and Packer [31] and Na^+^ concentration was determined using atomic absorption spectroscopy as described by Chen [32].

### Data analysis

Statistical analyses were carried out using the Statistical Product and Service Solutions (SPSS 19.0) software. All the parameters were compared using analysis of variance; the significance of fixed effects was tested with *F*-tests. Variation among lines in different time was analyzed by ANOVA according to Hansen and Roulund [33].

$$y_{ij}=\mu +L_{i}+T_{j}+\varepsilon_{ij}$$

where y_*ij*_ is the performance of an individual of line *i* within time *j*, *μ* is the overall mean, *L*_*i*_ is the line effect (*i* = 1,…,4), *T*_*j*_ is the time effect (*j* = 1,…,5) and *ε*_*ij*_ is the random error.

## Results

### Cloning of *TaLEA* and *ThbZIP* genes and obtainment of transgenic plantlets

**Fig 2:**
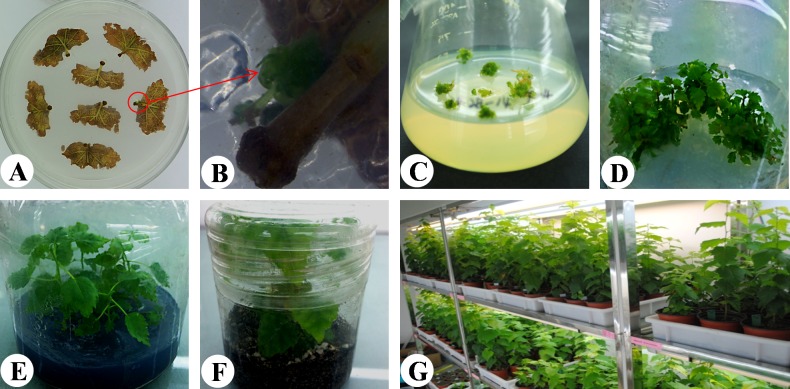
Regeneration of overexpressing *TaLEA* and *ThbZIP* transgenic birch. (A) The transgenic callus (red circle) have formed on one of the cut sites in a leaf segment. (B) Close-up view of the circled area in (A). (C) Some transgenic calls gained from leaf segments. (D) transgenic cluster of shoots has formed from a callus. (E) Four transgenic shoots were transferred to rooting medium. (F) One-month-old transgenic plants were grown in sterile soil media. (G) Three-month-old transgenic plants were grown in a greenhouse.

**Fig 3:**
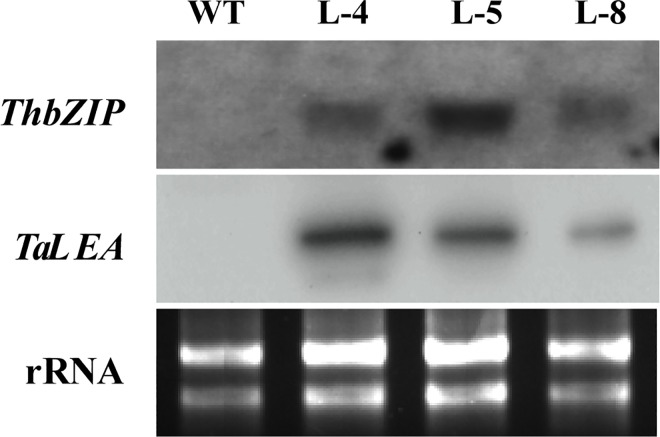
Northern blot analysis of *TaLEA* and *ThbZIP* expression in transgenic birches. Total RNA was extracted from the aerial tissues of one-month-old wild type and transgenic lines. The full length *TaLEA* and *ThbZIP* genes labeled with DIG-dUTP were used as probes. rRNA, ribosomal RNAs from different samples; *TaLEA*, target band of *TaLEA* gene; *ThbZIP*, target band of *ThbZIP* gene; L-4, 5, 8, three transgenic lines both containing *TaLEA* and *ThbZIP* genes; WT, wild type plantlet, respectively.

The *TaLEA* gene (GenBank accession NO.: DQ663481) was isolated from *T*. *androssowii*, which belongs to late embryogenesis abundant 3 superfamily protein. The *ThbZIP* gene (NO.: FJ752700) was isolated from *T*. *hispida*, a member of basic leucine zipper superfamily of plant G-box binding factor 1 (GBF1)-like transcription factors, which are involved in developmental and physiological processes in response to stimuli such as light, hormones or stress. To investigate the physiological functions of *TaLEA* and *ThbZIP* genes in birch, we produced transgenic birch lines ectopically overexpressing these two genes (Fig 2). In total, 11 independent transgenic lines regenerated on selection medium containing 50 mg L^−1^ Km. All transgenic birch lines were confirmed by PCR using specific primers for *NptII*. The positive control and transgenic lines all produced the expected 781 bp band (Fig 1B). Six transgenic lines contained only one of the genes (*TaLEA* or *ThbZIP*) and five contained both genes, as confirmed by PCR with specific *TaLEA* and *ThbZIP* primers (Fig 1C and 1D). A northern blot analysis confirmed that three of the transgenic lines (L-4, L-5 and L-8) had distinct bands corresponding to *TaLEA* and *ThbZIP* genes, while the wild type did not, confirming that the *TaLEA* and *ThbZIP* genes were successfully transcribed in mRNA level (Fig 3).

### Co-transfer of *TaLEA* and *ThbZIP* confers increased salt tolerance to transgenic tissue culture seedlings

**Fig 4:**
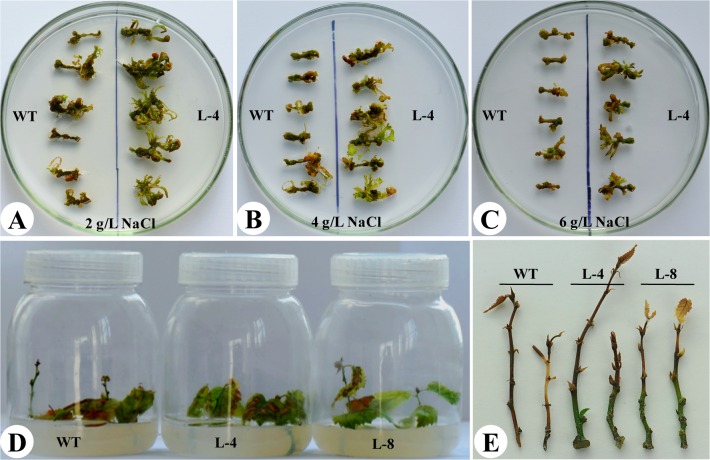
NaCl stress-tolerance test of transgenic birch ectopic overexpressing *TaLEA* and *ThbZIP*. Stems of transgenic and wild type plants were cultured on WPM medium containing 2 g L^-1^ (A), 4 g L^-1^ (B) or 6 g L^-1^ NaCl (C). Shoots of transgenic and wild type plants were transferred to 1/2 MS root medium containing 4 g L^-1^ NaCl (D), or 6 g L^-1^ NaCl (E). Photographs were taken 20 d following stress treatment. WT, wild type; L-4, transgenic line 4; L-8, transgenic line 8.

After the first 10 d, both transgenic and wild-type plants all started to produce callus differentiated from stem cut under 2 g L^−1^ NaCl stress, but transgenic stems had a stronger differentiation ability than that of wild type (Fig 4A). 20 d later, the number of adventitious bud in transgenic lines under 2 g L^−1^ or 4 g L^−1^ NaCl stress was prominently more than that in wild type (Fig 4A and 4B). Under the treatment of 6 g L^−1^ NaCl, transgenic stems could normally differentiate, but very slowly and the shoots were weak and withered. However, the wild-type stems barely produced adventitious buds after treatment, and finally died (Fig 4C). After 20 d, both transgenic and wild-type plantlets were hardly normally rooting under the treatment of 4 g L^−1^ NaCl, while the leaves of transgenic lines did not fall off and maintained green color (Fig 4D). With the increase in concentration of NaCl to 6 g L^−1^, wild type plantlets could not produce roots, then slowly dying, however, callus was still generated from the transgenic stem base, and the lower part of the stem was still in life activity (Fig 4E).

### Determination of a appropriate NaCl concentration for salt stress treatment

**Fig 5:**
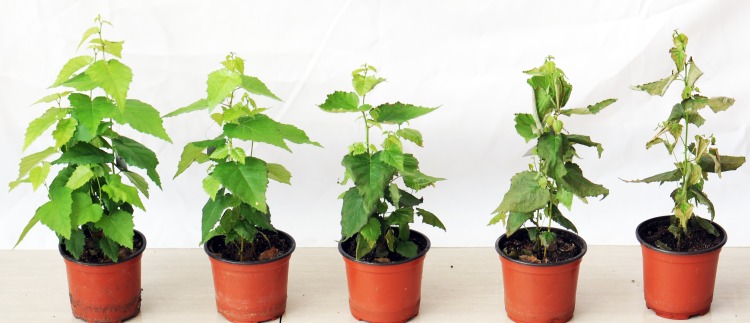
The phenotypes of WT on day 8 under different concentrations of NaCl. From left to right were WT, the treatment of 2 g L^-1^, 4 g L^-1^, 6 g L^-1^ and 8 g L^-1^ of salt stress, respectively.

Fig 5 shows the phenotypes of WT on 8 d under different concentrations of NaCl. The degree of damage increased as the salt concentration increased. In the 6 and 8 g L^−1^ NaCl treatments, the leaves were seriously wilted and the plants almost died. Plants in the 2 g L^−1^ NaCl treatment showed few salt stress symptoms, but those in the 4 g L^−1^ NaCl treatment showed wilting of a few lower leaves and most upper leaves on 8 d of the stress treatment.

**Fig 6:**
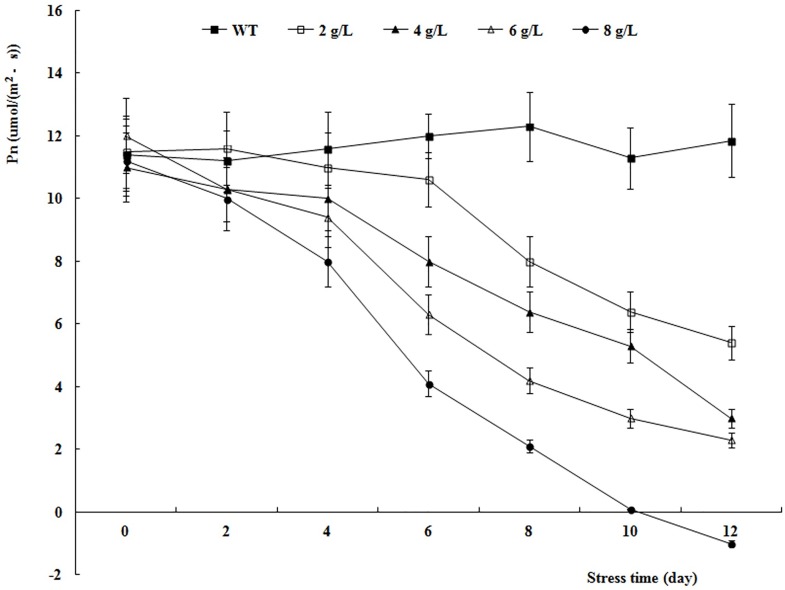
Pn values of WT under different concentrations salt stress.

**Fig 7:**
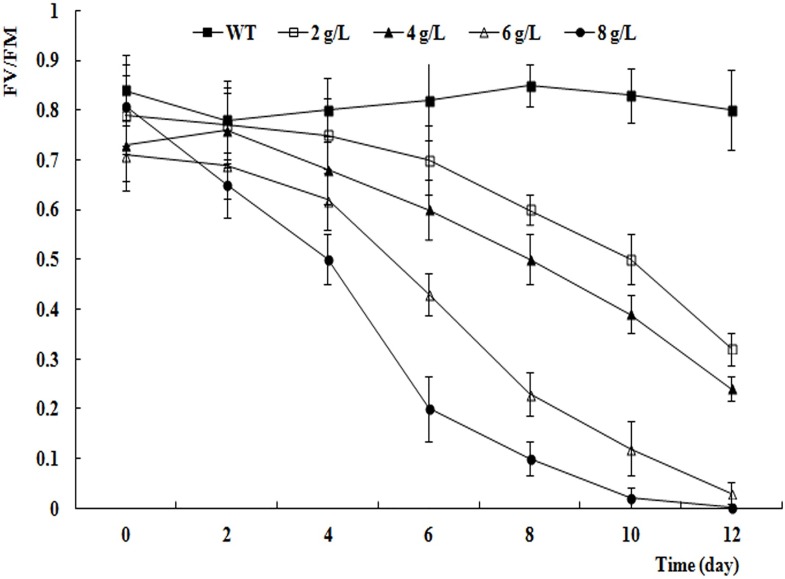
F_v_/F_m_ values of WT under different concentrations salt stress.

The Pn and F_v_/F_m_ values of WT decreased as the NaCl concentration increased and as the duration of the salt stress treatment extended (Figs 6 and 7). The Pn and F_v_/F_m_ values showed moderate decreases in the 4 g L^−1^ NaCl treatment, indicating that this concentration was appropriate to impose salt stress on birch.

### Pn–PPFD curves of different lines before and after salt stress treatment

**Fig 8:**
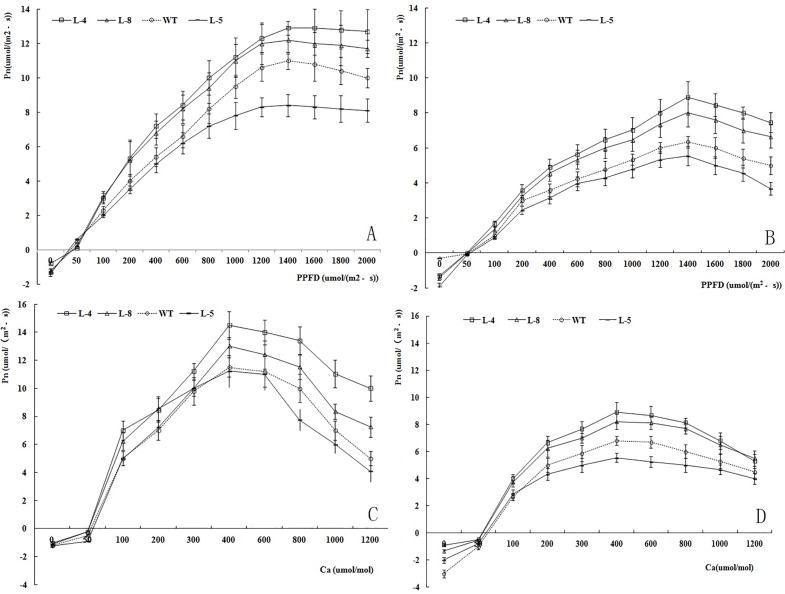
Pn-PPFD and Pn-Ca curves of different transgenic lines before stress (A, and C) and 8 d after salt stress (B and D).

The Pn–PPFD curves of the four different lines before salt stress are shown in Fig 8A. All four lines showed S-shaped curves. When the PPFD was zero, their Pn values were negative. The Pn values increased as the PPFD increased, with the maximum Pn value obtained at a PPFD of 1400 μmol m^−2^ s^−1^. The Pn values did not increase further, or even decreased a little, when the PPFD exceeded 1400 μmol m^−2^ s^−1^. At a PPFD of 1400 μmol m^−2^ s^−1^, the Pn values of lines L-4 and L-8 were higher than those of WT and L-5.

The Pn–PPFD curves of the four different lines after salt stress are shown in Fig 8B. As was the case before the stress treatments, all four lines showed S-shaped curves after the stress treatments. The Pn values increased as the PPFD increased to 1400 μmol m^−2^ s^−1^. In all of the lines, the Pn values at each PPFD were lower after the stress treatment than before the stress treatment. At each PPFD, L-4 had the highest Pn values and L-5 the lowest.

The Pn–PPFD simulation models before and after the stress treatments are shown in Table 1. The coefficients for all models were higher than 0.9, indicating that the models were effective. The LSPs of the four lines ranged from 1450 to 1630 μmol m^−2^ s^−1^ before the stress treatment and from 1175 to 1488 μmol m^−2^ s^−1^ after the stress treatment. The LCPs of the four lines ranged from 37.3 to 38.4 μmol m^−2^ s^−1^ before the stress treatment and from 50.0 to 68.9 μmol m^−2^ s^−1^ after the stress treatment. When the PPFD reached the LSP, the Pn values of L-4 were 12.9 μmol m^−2^ s^−1^ before the stress treatment and 8.9 μmol m^−2^ s^−1^ after the stress treatment, while those in L-5 were 8.4 μmol m^−2^ s^−1^ before the stress treatment and 5.56 μmol m^−2^ s^−1^ after the stress treatment.

**Table 1:** Pn- Par simulation equation and Pn, LSP, LCP values of different lines before and 8 d after stress.

Status	Line	Pn-PPFD simulation equation	Adjust coefficient(R^2^)	Pn (Max) (μ mol m^-2^ s^-1^)	LSP (μ mol m^-2^ s^-1^)	LCP (μ mol m^-2^ s^-1^)
Before stress	L-4	y = -0.000 005 *Par*^2^ + 0.0163 *Par* + 0.5424	0.9708	12.9	1630	44.4
	L-8	y = -0.000 005 *Par*^2^ + 0.0161 *Par* + 0.4416	0.9646	12.2	1610	42.9
	WT	y = -0.000 005 *Par*^2^ + 0.0145 *Par* + 0.0377	0.9780	11	1450	38.3
	L-5	y = -0.000 004 *Par*^2^ + 0.012 *Par* + 0.2175	0.9590	8.4	1500	37.3
After stress	L-4	y = -0.000 004 *Par*^2^ + 0.0119 *Par*—0.0212	0.9575	8.9	1487.5	50
	L-8	y = -0.000 004 *Par*^2^ + 0.0113 *Par*—0.14	0.9557	8	1412.5	50.4
	WT	y = -0.000 003 *Par*^2^ + 0.0088 *Par* + 0.1536	0.9583	6.34	1466.7	62.4
	L-5	y = -0.000 004 *Par*^2^ + 0.0094 *Par*—0.4981	0.9314	5.56	1175	68.9

### Pn–Ca curves of different lines before and after salt stress treatment

The Pn–Ca curves of the four lines before and after stress are shown in Fig 8C and 8D. The Pn values of the four lines were negative when Ca was zero, and increased with increasing Ca both before and after the stress treatments. The trends in Pn were the same among the different lines, but the maximum Pn value differed significantly (P<0.01) among the lines, ranging from 11.23 to 14.52 μmol m^−2^ s^−1^ before the stress treatment, and from 5.56 to 8.90 μmol m^−2^ s^−1^ after the salt stress treatment (Table 2). The CSP and CCP values were similar before and after the stress treatment in the different lines (Table 2).

**Table 2:** Pn- Ca simulation equation and Pn, CSP, CCP values of different lines before and 8 d after stress.

Status	Line	Pn-Ca simulation equation	Adjust coefficient(R^2^)	Pn (Max) (μmol m^-2^ s^-1^)	CSP (μmol mol^-1^)	CCP (μmol mol^-1^)
Before stress	L-4	y = -0.000 03 *Ca*^2^ + 0.0421 *Ca* + 0.3725	0.8832	14.52	701.74	50.04
	L-8	y = -0.000 03 *Ca*^2^+ 0.0389 *Ca* + 0.3517	0.8631	13.40	648.31	51.75
	WT	y = -0.000 03 *Ca*^2^ + 0.0374 *Ca*—0.1709	0.8903	11.53	623.30	54.50
	L-5	y = -0.000 03 *Ca*^2^ + 0.0358 *Ca*—0.0279	0.8224	11.23	596.77	57.55
After stress	L-4	y = -0.000 02 *Ca*^2^ + 0.0282 *Ca* + 0.107	0.8646	8.90	705.00	55.66
	L-8	y = -0.000 02 *Ca*^2^ + 0.0265 *Ca*—0.0877	0.8571	8.22	662.52	50.74
	WT	y = -0.000 02 *Ca*^2^ + 0.0251 *Ca*—1.0941	0.8323	6.82	627.56	54.19
	L-5	y = -0.000 02 *Ca*^2^ + 0.0191 *Ca*—0.4369	0.7892	5.56	655.45	55.63

### Differences among photosynthetic traits, antioxidant activity, and Na^+^ concentration among different lines under salt stress

**Fig 9:**
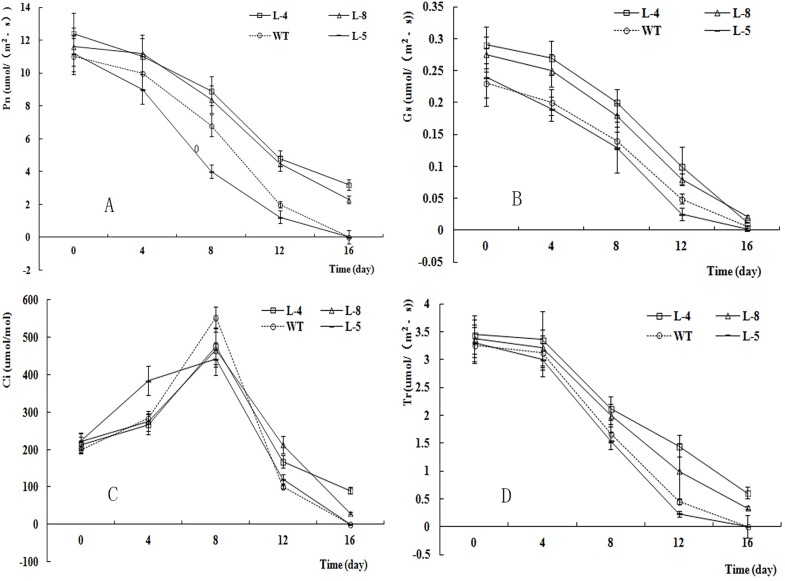
Pn (A), Gs (B), Ci (C) and Tr (D) values of different lines after different days salt stress.

**Fig 10:**
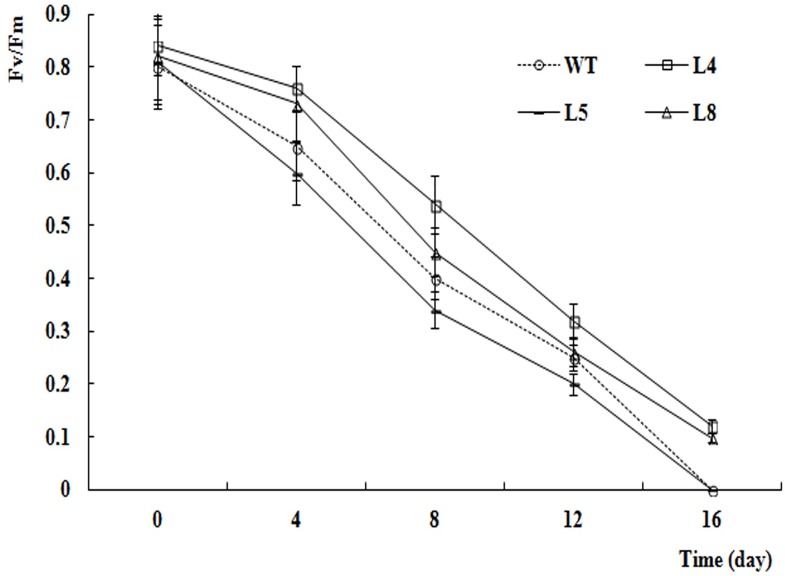
Fv/Fm values of different lines after different days salt stress.

The results of the ANOVA and *F*-tests of photosynthetic trait and F_v_/F_m_ data are shown in Table 3. All of the measured photosynthetic traits, including F_v_/F_m_, differed among lines and among sampling times. The trends in the photosynthetic traits of different lines are shown in Fig 9. After 16 d of salt stress, L-4 showed the highest Pn value and L-5 showed the lowest (almost zero, similar to that in WT). Similarly, the Pn–time curves, Gs–time curves, Tr–time curves, and F_v_/F_m_–time curves (Fig 10) of the different lines also decreased as the duration of the salt stress treatment extended. At each time point, L-4 showed the highest Gs, Tr, and F_v_/F_m_ values, and L-5 showed the lowest. In all lines, the Ci–time curve showed an upward and then a downward trend.

**Table 3:** ANOVA analysis of photosynthetic traits and Fv/Fm among different lines and time under salt stress.

Traits	Variance source	SS	df	Ms	F	P
Pn	Line	102.972	3	34.324	49.348	< 0.01
	Time	1049.768	4	262.442	377.313	< 0.01
Gs	Line	0.045	3	0.015	55.826	< 0.01
	Time	0.491	4	0.123	455.372	< 0.01
Ci	Line	59080.800	3	19693.600	14.233	< 0.01
	Time	70391.100	4	17597.775	12.718	< 0.01
Tr	Line	2.901	3	0.967	12.391	< 0.01
	Time	23.280	4	5.820	74.563	< 0.01
Fv/Fm	Line	0.334	3	0.111	27.492	< 0.01
	Time	3.078	4	0.769	190.070	< 0.01

Note: P < 0.01 indicated that there was significant difference between the homologous variation source.

**Fig 11:**
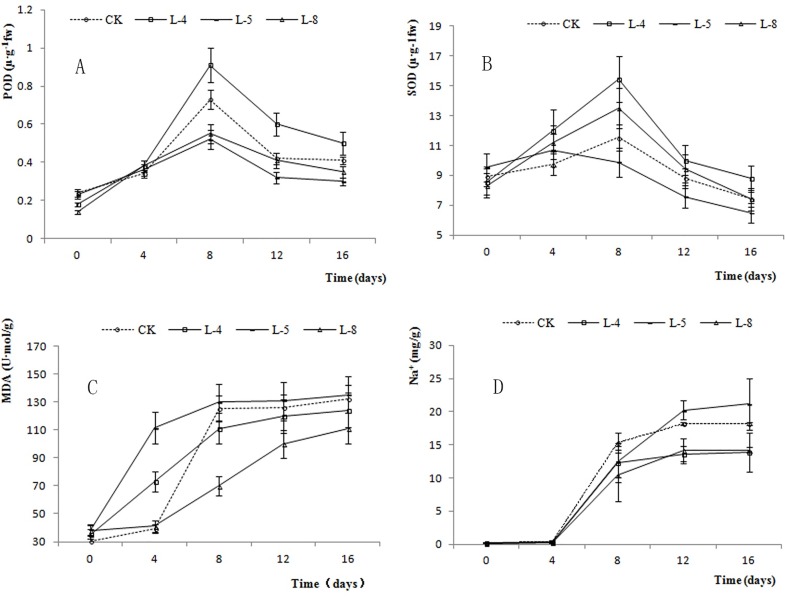
POD (A), SOD (B), MDA (C) and Na^+^ (D) values of different lines after different days salt stress.

The SOD and POD activities and MDA and Na^+^ contents differed significantly among lines and among different sampling times, as determined in the ANOVA and in *F*-tests (Table 4). Fig 11 shows the POD (Fig 11A) and SOD (Fig 11B) activities and the MDA (Fig 11C) content (Table 5) in different lines under salt stress. The POD and SOD activities first increased and then decreased under salt stress, while the MDA and Na^+^ contents increased in all of the lines. L-4 showed the highest POD activity among the four lines from 8 d to 16 d of the salt stress treatment. The SOD activity was also higher in L-4 than in other lines after 4 d of the salt stress treatment. Compared with the other transgenic lines, L-5 showed lower POD and SOD activities after 8 d of salt stress, and a higher MDA content. The Na^+^ content did not change markedly during the first 4 d of the salt stress treatment (Fig 11D), but increased markedly after this time. The Na^+^ content was higher in L-5 and WT than in L-4 and L-8 after 12 d of the salt stress treatment.

**Table 4:** ANOVA analysis of SOD, POD, MDA and Na^+^ Concentration among different lines and time under salt stress.

Traits	Variance Source	SS	df	MS	F	P
SOD	Line	38.474	3	12.825	8.491	< 0.01
	Time	189.441	4	47.360	31.357	< 0.01
POD	Line	0.228	3	0.076	9.518	< 0.01
	Time	1.729	4	0.432	54.093	< 0.01
MDA	Line	10372.813	3	3457.604	13.815	< 0.01
	Time	72087.441	4	18021.860	72.008	< 0.01
Na^+^ Concentration	Line	49.181	3	16.394	4.398	< 0.01
	Time	2555.841	3	851.947	228.564	< 0.01

Note: P < 0.01 indicated that there was significant difference between the homologous variation source.

**Table 5:** Average POD, SOD, MDA and Na^+^ content of different lines in different time under salt stress.

A	lines	0 d	4 d	8 d	12 d	16 d
POD (μ g^-1^ fw)	WT	0.24 ± 0.02 a	0.34 ± 0.02	0.73 ± 0.05 b	0.42 ± 0.03 b	0.41 ± 0.02 b
	L-4	0.18 ± 0.01 b	0.38 ± 0.03	0.91 ± 0.09 a	0.60 ± 0.06 a	0.50 ± 0.06 a
	L-5	0.23 ± 0.02 a	0.36 ± 0.03	0.52 ± 0.05 c	0.32 ± 0.03 c	0.30 ± 0.02 c
	L-8	0.14 ± 0.01 b	0.38 ± 0.03	0.55 ± 0.05 c	0.41 ± 0.04 b	0.35 ± 0.03 bc
	average	0.15 ± 0.09	0.36 ± 0.03	0.68 ± 0.17	0.43 ± 0.11	0.39 ± 0.06
SOD (μ g^-1^ fw)	WT	8.94 ± 0.67	9.72 ± 0.73	11.53 ± 0.87 bc	8.82 ± 0.66 ab	7.45 ± 0.56 ab
	L-4	8.58 ± 0.86	12.00 ± 1.20	15.45 ± 1.55 a	10.00 ± 1.00 a	8.78 ± 0.88 a
	L-5	9.54 ± 0.95	10.70 ± 1.07	9.88 ± 0.99 c	7.56 ± 0.76 b	6.49 ± 0.65 b
	L-8	8.32 ± 0.83	11.20 ± 1.12	13.50 ± 1.35 ab	9.43 ± 0.94 ab	7.40 ± 0.74 ab
	average	8.84 ± 0.86	10.91 ± 1.24	12.59 ± 2.42	8.95 ± 1.19	7.53 ± 1.05
MDA (U mol g^-1^)	WT	30.12 ± 2.26	39.40 ± 2.96 c	125.46 ± 9.43 a	126.07 ± 9.47 ab	132.17 ± 9.93
	L-4	35.35 ± 3.53	73.23 ± 7.32 b	111.00 ± 11.10 a	120.00 ± 12.00 ab	124.00 ± 12.40
	L-5	38.38 ± 3.84	111.50 ± 11.15 a	130.00 ± 13.00 a	131.00 ± 13.10 a	135.00 ± 13.50
	L-8	38.25 ± 3.83	41.11 ± 4.11 c	70.00 ± 7.00 b	100.00 ± 10.00 c	111.00 ± 11.10
	average	35.52 ± 4.56	66.31 ± 31.27	109.12 ± 26.23	119.27 ± 15.60	125.54 ± 14.00
Na^+^ (mg g^-1^)	WT	0.20 ± 0.13	0.39 ± 0.20	15.29 ± 1.55	18.13 ± 0.23	18.15 ± 0.23
	L-4	0.27 ± 0.05	0.23 ± 0.01	12.32 ± 2.91	13.64 ± 1.18	13.88 ± 2.91
	L-5	0.21 ± 0.04	0.39 ± 0.17	12.43 ± 2.40	20.25 ± 0.43	21.20 ± 3.88
	L-8	0.07 ± 0.04	0.22 ± 0.07	10.39 ± 3.88	14.15 ± 1.87	14.18 ± 0.43
	average	0.19 ± 0.10	0.31 ± 0.14	12.61 ± 3.02	16.54 ± 3.04	16.88 ± 2.91

Note: Different letters in a column are significantly different according Duncan's multiple range test and α = 0.05.

## Discussion

Abiotic stresses in plants involve a series of physiological and biochemical responses. A great many genes are associated with the abiotic stress-tolerance trait of plants, and multiple transcription factors were activated in different signal transduction pathways to respond single stress. As a result of the limited contribution of single gene to stresses, transfer of multiple transcription factors have been reported to produce additive or synergistic effects on stress tolerance in plants [34]. LEA proteins are a kind of multifunctional regulation proteins, which are closely related to the stress resistance of plants [35–37]. bZIP proteins are one of the most conservative transcription factor family, which are widely involved in the regulation of plant growth and development, and the response to various stresses [38–40]. As previous data, single gene (*TaLEA* or *ThbZIP*) and co-transfer (*TaLEA* and *ThbZIP*) both can enhance the salt and osmosis tolerance of transgenic tobacco plants [23–25].

Crossbreeding has been one of the most important traditional approaches to obtain new materials for tree breeding. However, this method is very time-consuming, because forest trees have long life cycles with extended vegetative phases ranging from one to many decades [41]. Genetic engineering offers the prospect of transferring desirable traits into selected genotypes at a comparatively faster rate by bypassing the reproductive process. The transfer of desirable trait(s) into forest trees by traditional approaches that involve breeding/recurrent selection would take decades, if not centuries, but, through genetic engineering, it can presently be accomplished in a single generation [42]. A number of genetically modified agricultural crops have been produced [43–44], but there are still many problems to overcome in the genetic modification of forest trees. Browning and vitrification during genetic transformation are two serious problems, because chlorophyll attrition affects transformation efficiency or even causes death [45–46]. In this research, activated carbon was added to the medium to reduce oxidation and minimize browning.

Determining the appropriate concentration of salt was the most important factor in the design of these experiments. Salt concentrations that were too high led to a rapid decrease in the photosynthetic index, which was not conducive to observation, and those that were too low did not affect the photosynthetic index sufficiently, or required a very long experimental period to detect any effects. In this research, the NaCl concentration of 4 g L^−1^ was determined to be appropriate; this is the same concentration that was used in a study on poplar [47].

Measurement of the Pn–PPFD curve is an important method to analyze the ability of plants to adapt to high or low light conditions [48]. In this research, the instantaneous Pn of all the lines increased with increasing PPFD when other environment factors were held constant, indicating that Pn was responsive to the illumination intensity. The LSPs ranged from 1450 to 1630 μmol m^−2^ s^−1^, indicating that birch can adapt to strong illumination intensity. The LSPs of transgenic lines were higher than that of WT, suggesting that the plants harboring *TaLEA-ThbZIP* showed greater resistance to high illumination intensity. After 8 d of salt stress, the Pn of the different lines increased slowly with increasing PPFD, and the LSPs and maximum Pns at the LSP were lower than those before the stress treatment. The results also showed that salt stress affected antioxidant enzyme activity and the Na^+^ content in plant cells, which ultimately affected photosynthesis, consistent with the results reported by Deng [49].

In the next 80 years, atmospheric CO_2_ concentrations are projected to double from the current concentration of 350 μmol mol^−1^ to 700 μmol mol^−1^. This increase will further stimulate plant growth and result in ecosystem changes [50] because CO_2_ is the most important substrate for photosynthesis. Within a certain concentration range, enhanced CO_2_ concentrations can promote the instantaneous photosynthetic rate [51]. There is strong evidence that plants have already responded to the increase in atmospheric CO_2_ concentration [52]. In this research, the CSP of the four different lines ranged from 596.77 to 701.74 μmol·mol^−1^. Below the CSP, the Pn of the four lines ranged from 11.23 to 14.52 μmol m^−2^ s^−1^, similar to the Pn values reported for poplar seedlings [28]. These results indicated that all four lines were able to utilize high concentrations of CO_2_. The instantaneous Pn under CSP was lower at 8 d of the salt stress treatment than before the stress treatment, indicating that salt stress affected photosynthesis in birch. The instantaneous Pn values were higher in L-4 and L-8 than in WT and L-5, indicating that the new genes had affected the physiology of the transgenic lines.

Chlorophyll fluorescence measurements are easy and rapid to conduct, do not damage plants, and sensitively reflect the relationship between the physiological status of the plant and the environment [53]. In recent years, chlorophyll fluorescence parameters have been used to measure resistance in many plant species [54]. In this experiment, the F_v_/F_m_ values differed significantly (P < 0.01) among the four lines and the five time points. The F_v_/F_m_ values of L-4 and L-8 were higher than those in L-5 and WT after 8 d of salt stress, indicating that the new genes had altered the efficiency of energy transfer from photosystem II, and increased the salt resistance of these lines.

During plant growth and development, many metabolic pathways produce reactive oxygen species, which can damage cell membranes and lead to cell death [55]. During evolution, plants have evolved an antioxidant enzyme system (including SOD and POD), which removes reactive oxygen species, prevents membrane lipid peroxidation, and maintains normal plant growth and development [56]. The intermediate produced during lipid peroxidation is MDA. If lipid peroxidation continues unchecked, it can result in structural damage to cell membranes and cell death [57]. In this research, as the duration of salt stress treatment was extended, SOD and POD activities first increased and then decreased, suggesting that the plants were able to remove free radicals via increased SOD and POD activities at an early stage. As the salt stress treatment continued, the POD and SOD activities decreased, likely because of damage to various cellular functions. The increased in MDA content during the salt stress treatment suggested that cell membranes were destroyed progressively, ultimately leading to plant death. After the salt stress treatment, the SOD and POD activities were higher in L-4 than in WT, while L-8 had higher SOD activity but lower POD activity than those in WT. In contrast, after 8 d of salt stress, the MDA content was higher in L-5 and WT than in L-4 and L-8, indicating that the new genes enhanced the antioxidant capacity of L-4 and L-8 but reduced that of L-5. The result showed that inserting exogenous genes markedly affected plant growth and development. Previous studies have found that, high expression level of *GmbZIP78* leads to not only reducing salt resistance, will also affecting plant growth [58]. Overexpression of *bZIP* genes in *Arabidopsis thaliana* (*ABF1*, *ABF2*, *ABF3*, and *ABF4*) leads to slow growth, dwarf, and abnormal phenotype [59]. That is, *bZIP* may exist the most appropriate concentration range *in vivo*, too high may lead to the imbalance of transcriptional regulation.

The ability of a plant to tolerate salinity is related to its ability to maintain ion homeostasis in cells. Many different soluble salts can reduce the osmotic potential of plant rhizosphere, making it more difficult for the plant to absorb water, leading to physiological drought [60]. The Na^+^ content in leaves indirectly reflects the amount of Na^+^ absorbed by the roots, and is an indicator of the degree of stress. In this study, as the duration of the salt stress treatment extended, the Na^+^ content in leaves of all lines increased to different degrees, indicating that salt stress affected the growth of the lines differently. The average Na^+^ content showed little change after 4 d of salt stress, but had increased markedly after 8 d of salt stress. The Na^+^ contents were lower in Lines L-4 and L-8 than in WT and L-5, indicating that the former two lines were more salt-resistant than the latter two lines.

## Conclusions

With the development of science and technology, increasing numbers of transgenic varieties of various crop species (maize, cotton, soybean, canola, squash, papaya, alfalfa, and sugarbeet) have been produced [61]. In forest research, many genes have been inserted into the genomes of various tree species to accelerate growth, improve wood properties, and confer resistance to environmental stresses [62–63]. All of these quantitative traits are controlled by hundreds of genes, and are thus unlikely to be substantially affected by the transfer of only one or two genes. However, recent research has suggested that some quantitatively inherited traits can be substantially modified by altering just one gene by genetic engineering [64]. In our study, constructs containing both *TaLEA* and *ThbZIP* genes were transformed into birch. Because the transcriptional level of these exogenous genes differed among the lines, the various transgenic lines showed different physiological properties under salt stress. Lines L-4 and L-8 were selected as excellent lines because of their strong salt resistance. Further research should characterize their growth and stem traits, and the regulation mechanism of the exogenous genes. Also, we should fully consider the safety of releasing and cultivating transgenic plants in the field.

## Supporting Information

S1 Table All primers used in this study. (DOCX)
