# Supplementary material for: Variation Analysis of Physiological Traits in Betula platyphylla Overexpressing TaLEA-ThbZIP Gene under Salt Stress
Source: PLoS One. 2016 Nov 1;11(11):e0164820. doi: 10.1371/journal.pone.0164820 (PMC5089751; doi:10.1371/journal.pone.0164820)
Supplement: S1 Table — (DOCX) [file pone.0164820.s001.docx]

| **S1 Table.** **All primers used in this study.** | |
| --- | --- |
| Name | Sequence |
| *NptII*-F | 5'-AACAAGATGGATTGCACGCAGGTTCTCCGG-3' |
| *NptII*-R | 5'-GAACTCGTCAAGAAGGCGATAGAAGGCGAT-3' |
| *TaLEA*-F | 5'-ATGGCTCGCTGCTCTTACTC-3' |
| *TaLEA*-R | 5'-TCAGTGAGAGGATCGATTGAA-3' |
| *ThbZIP*-F | 5' -ATGTATCAACCCGTGAGTTC-3' |
| *ThbZIP*-R | 5' -TTAGAACTGAAACATATCAG-3' |
